# FTO2/438: Implementation of a Telematics System for the Management of Epidemic Emergencies

**DOI:** 10.2196/jmir.1.suppl1.e27

**Published:** 1999-09-19

**Authors:** S Bellini, P Colangeli, E Isocrono, A Giovannini, C Di Francesco, V Caporale

**Affiliations:** ^1^Istituto Zooprofilattico Sperimentale dell'Abruzzo e del Molise, TeramoItaly

**Keywords:** Telematic System, Disease Outbreak Management, Epidemic Emergencies;

## Abstract

**Introduction:**

A system to support decisions and operations in cases of epidemic emergency has been designed and implemented, in order to improve the decision-making capabilities of Veterinary Services for outbreaks of exotic diseases.

**Methods:**

The system implementation consisted of: 1) drafting contingency plans for OIE List A diseases; 2) implementing an automated information network, linking Local Veterinary Unit and the Regional Epidemiological Centre; 3) implementing a Geographical Information System (GIS), to be automatically connected to the animal identification database and to the ANIMO (Animal Movement) system; 4) supplying the personnel of Veterinary Services with the necessary tools, instruments and materials; 5) personnel training. Integration of activities led to the implementation of a telematic support system for the management of epidemic emergencies, providing the Veterinary Services with the information necessary to the management of exotic disease outbreaks. The system has been implemented from a structural point of view as follows:

1. Data warehouse design and implementation, fed by ORACLE DATA MART SUITE operational databases,
2. Implementation within the Istituto Zooprofilattico Sperimentale dell'Abruzzo e del Molise Web site, of a controlled access telematic system, where:
  - static pages were implemented in HTML and the dynamic ones in PERL;
  - GIS was used to design and update maps;
  - Downloading of documents and forms was made possible as well as the generation of tables and graphs in real time.

**Results:**

In the event of an outbreak it is possible to: map relevant data and information (i.e. Protection and Surveillance Zones); to produce disease trend data both in tabular and graphical form and the indicators for the disease management and control. Contingency plans of OIE list A disease are provided through the Internet for consultation and downloading. All the forms for administrative and epidemiological data collection are provided for and can sent by e-mail to the proper veterinary authority and other stakeholders.

**Discussion:**

The system has been tested both by a simulated foot and mouth disease outbreak and a real Swine Vesicular Disease outbreak. The existence of written and standardised procedures, the availability of updated and pertinent information for outbreaks management and the support of a telematic system has allowed the rationalisation the actions to be implemented and to speed up intervention time.

